# Psychological and physical effects of breast cancer diagnosis and treatment on young Ghanaian women: a qualitative study

**DOI:** 10.1186/s12888-020-02760-4

**Published:** 2020-07-06

**Authors:** Merri Iddrisu, Lydia Aziato, Florence Dedey

**Affiliations:** 1 Department of Adult Health, School of Nursing and Midwifery, University of Ghana, P. O. Box LG43, Legon, Accra, Ghana; 2 School of Medicine and Dentistry, University of Ghana Medical School, Accra, Ghana

## Abstract

**Background:**

Young women diagnosed with breast cancer face challenges that interfere with their entire life with psychological and physical effects.

**Method:**

We employed a qualitative exploratory descriptive design, and recruited twelve participants through purposive and snowball sampling methods to conduct 12 face to face individual interviews. A reputable review board in Ghana; Noguchi Memorial Institute for Medical Research, gave ethical clearance for data collection. Data were transcribed verbatim and analysed using thematic content analysis.

**Results:**

Three themes emerged from the data; physical effects of breast cancer, effects of treatment on body image, and emotional effects of breast cancer diagnosis and treatment. The negative effects of treatment incapacitated most of the women and limited their activities of daily living. Some experienced severe bodily weakness and stayed indoors for days. Most participants felt they looked unattractive because they have had a mastectomy done, and used pieces of rags and handkerchiefs as breast prostheses. Those who had hair loss through chemotherapy used different kinds of wigs to cover their baldness. Almost all the participants cried, felt depressed, and became emotionally unstable when they were told their diagnosis. Some denied their diagnoses and displaced their anger on healthcare personnel. A few of them felt they had brought disgrace to their families because breast cancer is perceived, a disgraceful disease.

**Conclusion:**

Young women diagnosed with breast cancer require psychological interventions and physical support from healthcare personnel and their families.

## Background

Breast cancer is the second most common cancer after cervical cancer in Sub-Saharan Africa [1, 2]. In African women, it is known that breast cancers are diagnosed at earlier ages than in high-income countries [3] with Sub-Saharan Africa recording the highest incidence [4]. Breast cancer in young women is mostly hormone-receptor-negative, aggressive with limited treatment options, and has a poorer prognosis than that of older women [5].

In Ghana, breast cancer is the most prevalent cancer, and one of the commonest causes of cancer deaths in females of all ages with an alarming incidence in young women aged 40 to 49 years [6]. Der Muonir, Naaeder, Tettey and Gyasi [7] reported that breast cancer affects a higher proportion of young women with 47–57% of cases seen in women less than 50 years. It was reported during the first Global Health Workshop on cancers held in Accra (Ghana) in the year 2015 that, cases of breast cancers continue to rise especially, among young women aged 25 to 30 years. In the year 2018, 36.3% new cases of breast cancers were estimated to occur in Ghana with 12.4% deaths [8].

The diagnosis of breast cancer comes as a surprise to every woman no matter the age or social status because women perceive their breasts as something that makes them whole or complete, and therefore feel demoralized and useless when they lose their breast [9, 10]. The breasts of women serve several purposes including nourishment for their offspring, an erotic organ in a relationship, and a symbol of being feminine [1]. When a woman is diagnosed with breast cancer, she becomes hopeless, tearful, ashamed, and discouraged because of society’s behaviour towards breast cancer patients [11]. Depression is reported to characterize the diagnosis of breast cancer with a sense of helplessness, hopelessness, and a lack of motivation to effectively cope with life [12]. Young women living with breast cancer have more severe depression than older women [13]. These women have physical, psychological, and social concerns that require special care from a multidisciplinary healthcare professional [14, 15]. Even though breast cancer comes with a myriad of challenges, studies indicate that when people are diagnosed with breast cancer, they gain personal strength out of their experiences, and become psychologically confident, emotionally mature, appreciate life, get closer to God, develop empathy for others and intimacy for family [16, 17].

Treatment of breast cancer generally involves chemotherapy, radiotherapy, and surgical intervention [18, 19]. While surgery leaves a deformed breast and a scar tissue that women find demeaning, chemotherapy and radiotherapy come with weight loss, vomiting, weakness, loss of hair, and skin discolorations that distract their daily activities [20]. Hormonal therapy also brings about; hot flushes, muscle cramps, joint stiffness, joint pains, and loss of libido [21], which may be disturbing for a young woman to go through, since that is regarded as something that would have been experienced in her old age. Physiologically, breast cancer treatment comes with sexual and fertility concerns, including; diminished desire for sexual intercourse, fear of sexual intimacy, menstrual irregularities, and destruction of ovaries resulting in infertility [22, 23].

Breast cancer treatment, therefore, affects young women’s physical appearance, sexual and reproductive lives as well as employment [24–26]. Even though breast cancer mostly affects older women, the literature indicates that some Ghanaian young women suffer breast cancer and there is a paucity of literature on the experiences of young women with breast cancer in Ghana that could help health workers understand and care for them better. This study, therefore, explored the physical and psychological experiences of young women with breast cancer in the Accra metropolis. The study was undertaken as part of the first author’s master’s degree programme at the University of Ghana.

## Methodology

The study employed an exploratory descriptive approach to qualitative research to explore, understand, and document the experiences of young women with breast cancer. The design was considered suitable since little is known about the phenomenon [27].

### Study setting

The study was conducted in the Accra Metropolis, Ghana, and participants were recruited from three hospitals: a regional hospital, a quasi-government hospital, and a tertiary teaching hospital that serves as the United Nations Level Four Medical facility.

Women in their fertile age 15–49 years [28] were the target group for the study but the ages of those interviewed, ranged from 28 to 45 years [17]. These women had all been diagnosed with breast cancer (advanced or early) and had undergone some form of treatment. Breast cancer patients who were newly diagnosed (3 months and below), those very ill and on admission, and those who were with obvious mental health problems, were excluded.

### Sample size and sampling technique

The sample size was 12 based on the attainment of saturation. Saturation is the point when no new responses or repetitive responses are obtained from study participants [27]. Purposive and snowball sampling techniques were used to select participants who provided more in-depth information on their (lived) experiences with breast cancer [29].

### Data collection procedures

The researchers obtained ethical clearance from the Institutional Review Board of the Noguchi Memorial Institute for Medical Research, University of Ghana. A permission letter was attached to copies of the ethical clearance and sent to the three health facilities selected. The first author visited the selected facilities to work with the staff at the breast clinics and the in-patient units briefly before data collection began for participants to be familiar with her. During the interaction, the first author made it known to participants that she is a nurse clinician who was in school pursuing a masters’ programme and has ever taken care of breast cancer patients. The first author gave her contact number to some of the Staff of the selected facilities and asked them to help recruit participants for the study. The authors sought informed consent from every participant and explained to participants that they had the right to withdraw from the study at any time without any consequences. Participants were also given clear unambiguous information regarding the study and were assigned pseudonyms and identification numbers which were used throughout the study to increase confidentiality. Contact numbers of participants were obtained and calls made to schedule an interview with those who consented to participate in the study. The first author conducted the interviews individually at the convenience of participants in English, in their homes, in a room at the premises of the health facilities, and at a place of choice closer to participants’ neighbourhood. A semi-structured interview guide was used during interviews, the interviews lasted 35 to 60 min. During interviews, the researcher was careful not to project her experiences on the participants and wrote down exactly what participants portrayed. Responses of participants were probed further where necessary to allow them to describe their experiences fully. The interviews were tape-recorded, an approach which increased the accuracy of the descriptions of participants’ experiences and consequently increased the credibility of the findings. The researchers considered at all times, stringent attention to details, adhered to procedures, and ensured consistency and accuracy.

### Data analysis

Data analysis was done alongside data collection. The recorded interviews were transcribed verbatim and read several times to understand exactly what the participants said by two independent coders. First, codes were assigned to meaningful chunks after carefully getting the essence of the data. Similar codes were cautiously grouped to form subthemes and subsequently, themes. The researchers kept logs for reviews to ensure that participants’ views were truly represented. Discussions were done among the researchers on the themes generated, and ensured that the data were free of personal biases. The themes were then put under the various objectives set for the study; physical and psychological effects of breast cancer diagnosis and treatment. The data was finally exported to NVivo version 11 which was used to manage the data. Thick verbatim quotations were also used to back the findings of the study which brought alive the true experiences of the young women.

## Results

### Sociodemographic Characteristics of participants

The ages of the participants ranged from 28 to 45 years and among the twelve participants, only one had a family history of breast cancer. The ethnic backgrounds of participants include; Ga, Ewe, Akuapem, Ga-Adangme, and Ashanti which made the data very rich.

Table of Sociodemographic Characteristics of participants

**Table:** 

Identity	Marital status	No of children	Level of education	Time since diagnosis	Treatment received
YWP1	Married	3	Tertiary	4 yrs.	Mastectomy, Chemotherapy
YWP2	Single	2	Tertiary	1 ½ yrs.	Mastectomy
YWP3	Divorced	1	J. H. S	2 yrs.	Chemotherapy
YWP4	Married	2	Tertiary	7 months	Mastectomy, Radiotherapy
YWP5	Single	1	Tertiary	10 months	Chemotherapy
YWP6	Single	None	S. H. S	11 months	Chemotherapy, Hormonal therapy
YWP7	Single	1	Tertiary	8 months	Chemotherapy
YWP8	Married	4	S. H. S	1 ½ yrs.	Mastectomy, Hormonal
YWP9	married	2	J. H. S	9 months	Chemotherapy
YWP10	Single	None	Tertiary	8 months	Alternative treatment Mastectomy
YWP11	Married	1	Tertiary	1 ½ yrs.	Chemotherapy
YWP12	Married	4	S. H. S	4 yrs.	Mastectomy Chemotherapy Radiotherapy

The three themes that emerged include: physical effects of breast cancer, effects on body image, and emotional effects of breast cancer diagnosis and treatment.

### Physical effects of breast cancer on young women

Young women in this study complained of their inability to carry out their activities of daily living because of the effects of medications given to them. They could not walk, eat, or drink water after chemotherapy sessions. Some had their treatment suspended for a while because of severe anaemia.

*“ … initially when I went through the chemo I did not take it easy at all, I had a weakness, I vomited continuously for three days and could not eat or drink anything. When I went for the next session, the doctor said I did not have enough blood in me so he suspended the chemo and wrote blood tonic for me to go and buy”* (YWP8).

*“I remained indoors because I was weak and I could not bath, walk or eat so I refused to go for the fifth one as scheduled* (YWP10).

The side effects of the treatment also negatively affected participants’ social and spiritual life, comfort, and care of their children due to constant severe headaches.

*“I have constant severe headaches, and my immune system is also down. When I started the chemotherapy, it took me about three weeks in the house battling with the effects without being able to go to work. I vomited and became very weak so I was unable to pray”* (YWP5).

“*… when I go for the chemo, I am not able to cook and also care for my children. I vomit when I smell food”* (YWP9).

### Effects of breast cancer treatment on body image

Participants who had chemotherapy done, had their hair falling off which they considered a great loss because of how admirable their hair is.

*“I have lost my long flamboyant hair, the long hair I decided to hold and twist anyhow, hmm. People use to admire my hair a lot when I go to the salon* and now m*y husband has been complaining about the loss of my long beautiful hair”* (YWP11).

*“I have lost all my hair when I combed it two days after the chemotherapy, my hair came off so I shouted. My auntie became alarmed and reshaped it for me but later, everything came off rendering me bald*” (YWP6).

Participants relied on wigs both human and synthetic to make up for their hair loss. Some purchased different types of human hair to change their looks.

*“Now this wig is what I have to live with until a miracle occurs. I have bought three types of human hair to appear attractive and be able to go to work”* (YWP7).

*“My husband complains about the wig and said I should not wear the wig but I cannot live without the wig, I just don’t feel comfortable at all seeing my scalp bald”* (YWP9).

Darkening of the tongue, gum, skin, and the nails as side effects of chemotherapy became so obvious in some participants to the extent that, one participant likened herself to a smoker. She further narrated about her breast shrinking and discharging.

*“Hmm! look at my colour, I have generally darkened including my tongue and gum. Just have a look at my nails, you might think I smoke a cigarette and my breast too is shrinking and discharging, hmm life”* (YWP7).

Participants who went through mastectomy complained about their inability to wear brassieres when dressing up because of their wounds and the pains associated with it. This made it difficult for a participant to go out because she found it embarrassing when her nipples pointed out in her dress when she was not wearing a brassiere.

*“with the big plaster on my wound, I cannot go out. My wound was as big as my palm or yours but though it is getting smaller, I cannot wear a bra because of the pain and also my nipples point out in my dress without a bra and it is embarrassing to me”* (YWP1).

The loss of the breast through a mastectomy affected participants’ dressing and choice of dresses. Aside, some developed lymphoedema with its associated pain and discomfort which affected their quality of life.

*“After the surgery, something like a boil developed in my armpit which is painful and because of that I do not wear sleeveless dresses anymore”* (YWP12).

*“My challenge now is that I like wearing sleeveless but now that I have lost one breast, my only choice is to wear dresses to cover my chest completely. I have small breasts so I wear a bra that pushes up my breasts but now how can I do that again? Hmm!”* (YWP4).

In the absence of the breast, some participants folded rags, handkerchiefs, and gauze and put these in their brassier as breast prosthesis. Others emphasized that the non-availability of silicone breast prosthesis made them waste a lot of time when dressing up. Even for participants who have not undergone mastectomy but have had their affected breasts shrunk, used handkerchiefs as a prosthesis to augment the size of their breast.

*“I fold soft rag and put in my bra to replace my breast before dressing up. I have searched for the original breast prosthesis to buy but it is difficult to get. It makes me stand in front of the mirror for a long time.”* (YWP2).

*Oh! it is not easy my sister, after the surgery, I place abdominal pack in my bra and it looks just like my breast“* (YWP10) “*… I spend a lot of time dressing up because I have to make sure the breasts are of the same size. I hope to get an artificial breast someday.”* (YWP12).

“*Even though my breast has not been removed, it has shrunk and my dresses do not fit me anymore, now when I am dressing, I put a handkerchief on it before I move out of the room. I do it with care to achieve the same breast size.”* (YWP11).

There were also changes in body sizes due to the effects of chemotherapy. While some complained of reduced body sizes, others alleged they put on weight due to the treatment received.

“… *I received a lot of intravenous fluids during my chemotherapy so I have grown fat and my dresses cannot fit me anymore. I also went through axillary clearance which has also resulted in lymphoedema so I dress strategically now due to my health.”* (YWP10).

*“This disease has … come to spoil a lot of things. Physically I have changed****,****I used to be fatter than this but I have grown lean now. I am not able to wear my dresses anymore because they are now loose on me.”* (YWP9).

### Emotional effects of breast cancer diagnosis and treatment

Most participants in the study did not receive enough counseling during their diagnosis, as most of them were shocked, sad, and cried because they never thought they could develop breast cancer one day. This further intensified with unkind reactions from hospital staff.

*“I was not given anyone to talk to me no, the doctor told me I have breast cancer and I shouted, Jesus! He looked at me and said go and think of it so that you come for us to take off the affected breast”* (YWP 8).

*“After the tests, the doctor finally said ‘Madam I am afraid you have breast cancer’ and said I have to go through treatment. I was annoyed because when I detected it and reported, they only gave me antibiotics on two occasions so I waited for a year before they saw it, just imagine this”* (YWP 12).

“*… I cried, I cried to the extent that all the nurses came to surround me. Oh, I never thought I could get breast cancer one day, not at all, that is the last thing to cross my mind. I never thought of it so I was shocked and sad”* (YWP9).

*“I cried, I really cried that day, and my Aunt tried consoling me but I did not even pay attention to what she was saying. I kept asking why me! Why me?”* (YWP6).

A participant said she could not sleep, eat or think for a week or two and saw her world crashing before her.

*“Oh I was shocked, I was shocked because we do not have such a disease in our family. There is no diabetes or hypertension in our family and I have not also heard about this kind of disease in our family before so I was shocked”* (YWP5).

*“It was not easy for me at all, and I did not take it lightly … I realised that I was in trouble and I asked myself wherefrom this? For about a week or two I was not myself. I could not sleep, eat or think properly and I saw my world crashing before me*” (YWP7).

Some participants lamented they became mentally unstable and started talking to themselves, dressed shabbily, moved out of their houses, and went roaming. Others felt they had brought a disgrace to their families because breast cancer is a disgraceful disease.

“*One day I was sitting on a bus talking to myself. I did not know where I was going until the bus attendant asked me where I was alighting. It was then I realised I neither had a destination in mind nor money to pay for, meanwhile, my baby was at my back. Hmmm I nearly got mad”* (YWP4).

***“****When it started, I nearly got mad because I could wear any dress at all without considering where I was going.”* (YWP6).

*“Among my siblings, I am the only one suffering from breast cancer, … so I feel I am the first person to have brought disgrace to our family hmmm!”* (YWP9).

A participant narrated she denied the news of her diagnosis and remained indifferent when she was told her diagnosis in the consulting room. She left the consulting room and asked her mother to get up for them to go home.

*“ … the doctor asked … if I heard him and I said yes, you said I have breast cancer and I heard you perfectly. I just got up, picked up my bag, and asked my mother to get up so that we could go home. I was in denial but later the reality hit me”* (YWP3).

One participant refused to eat or talk to people around her while another continued to cry because she did not understand why she suffered breast cancer.

“*… I woke up with so many tears and sobs, I don’t know, it just came I don’t know why it should happen to me. I don’t know the reason behind it and I don’t know what I have done … I was in real emotional something especially when I saw that the breast wasn’t there* (hmmm)” (YWP3).

“*… when the wound was opened the third day after surgery, oh my God! That was when I realised my breast was gone. … I was annoyed so I stopped talking to all the nurses, I was just crying... In the beginning, it was like a dream but now I have seen that it is real”* (YWP4).

Fear of death was also an issue some participants had as they contemplated what would happen to their husbands and children should they die from breast cancer.

*“I fear that I may die and leave my husband and children. Though the nurses have talked to me, I’m still afraid I may die for someone to come for my husband and maltreat my children* (shedding tears)” (YWP9).

## Discussion

The study found that most participants after receiving chemotherapy went through the usual side effects of chemotherapy as reported in [30, 31]. These effects negatively impacted their activities of daily living, impaired participants’ work, and household chores including care of their children and prayer life. On the contrary, some women rather use activities to keep themselves busy so that they do not concentrate on the side effects [32]. How participants narrated their ordeal implied, they were not adequately prepared and educated on the side effects of treatment before they began treatment. Women in Northeastern Thailand who were prepared psychologically, physically, and socially before treatment, had no such problems with side effects [33]. However, a study indicated that some cancer patients also refuse to do their routine work because of fear of exerting pressure on their bodies, but not because of the effects of medications [34].

Body image concerns raised by participants, for example, hair loss, absence of the breast that was complicated by unavailability of breast prosthesis, presence of lymphoedema, and skin changes, affected participants’ quality of life. Similarly, findings from earlier studies confirm that changes in body image among women with breast cancer cause emotional disturbances that permeate their thinking, particularly when looking into the mirror, dressing up, taking a shower, and during sexual intimacy with their partners [35, 36]. Meanwhile, behavioural therapy and exercises are found to offer long term solutions to cancer-related treatment side effects [37].

In the absence of breast prosthesis, women in this study used rags, handkerchiefs, and pieces of gauze in their brassieres as breast prostheses after mastectomy. This is in tandem with findings from [38] where women use cotton and pieces of cloth in their brassieres after mastectomy. This is a common practice among women in Sub-Saharan Africa where breast reconstruction surgeries are limited, and also, breast prostheses are difficult to procure and afford by people. However, in the advanced countries, even though women who undergo a mastectomy have access to silicone breast implants and other breast reconstruction techniques, these women equally encounter pain and poor appearance of their reconstructed breast with some opting to maintain a ‘flat chest’ to avoid multiple surgeries and risks of silicon related dangers [39, 40]. There is a need for health care providers to procure image enhancement equipment like silicone implants to give hope to those undergoing surgery. There should also be avenues for clinical psychologists to give care and do follow-ups on patients to support them psychologically.

Miller, Gorcey, and McLellan [24] indicated that chemotherapy causes skin and nail changes that negatively affect women’s self-esteem and bodily functions which is affirmed by another study [41]. Equally, some participants in this study experienced obvious darkening of the tongue, gum, skin and nails, and weight changes after receiving chemotherapy. Also, some participants lost their hair and resorted to using wigs as a cover. Studies indicate that alopecia and decreased body weight are treatment effects people experience, with alopecia being the most disturbing side effect [38, 42]. On the contrary, in a study done among Muslims, participants did not worry about hair loss because it is not something permanent, as they prefer the wearing of a scarf to a wig [38]. This could possibly be because of the participants’ religious affiliation unlike that of the current participants.

Interestingly, the weight gain was not much of a problem to participants who experienced it, just that their old dresses were not fitting them anymore. This was not surprising because typical African women cherish weight gain and body fat as a sign of good living.

Emotionally, when participants were told their diagnoses, most of them were stunned, became sad and cried because they had no family history of breast cancer. A study indicates that fear, loss of hope, emotional instability, shock, suffering, and guilt characterise breast cancer diagnosis and treatment [43]. Some of the participants’ emotions intensified because of pending surgeries, and body image changes they observed with chemotherapy. A few of the participants could not sleep, eat, or reason for days while others refused to eat or talk to people around them [44]. This is part of a normal grieving reaction that could be exhibited by anyone experiencing a loss [45]. To affirm this, an earlier study found that being diagnosed with breast cancer could be equated to a hard knock on the head, a bomb blast, or a sudden tragedy, with a sense of humiliation, hopelessness, sleeplessness, and reduced bodily strength [46]. This could also be because of the perception people have about breast cancer as an evil disease [47]. While some participants went into denial, a few dressed shabbily and roamed in reaction to their diagnoses. Even though this corroborates findings from earlier studies [32, 48, 49], some participants’ reactions were to the extreme. In Ghana, clinical psychologists are few in the health sector and are found mainly in the teaching hospitals and some regional hospitals. Usually, a medical doctor has to refer a patient before the person can access the services of these clinical psychologists. If the participants in this current study were to receive enough psychological care, they would not have reacted the way and manner some did. Meanwhile, this is not the case in the advanced countries where patients can request for the services of a clinical psychologist when the need arises [50].

It is widely recognised that management of treatment-related complications is a key component of supportive care, which has a well-defined clinical guideline but experts are of the view that this part of the breast cancer treatment is likely to be overlooked in Low, and Middle-Income Countries like Ghana [51, 52]. From the finding of this study, it is evident that supportive care is not fully implemented in Ghana’s healthcare system. It is therefore important for these guidelines to be followed in breast cancer care in Ghana. The ministry of health and the Ghana health service could adopt and implement supportive care programs outlined by the panel of experts and devote resources to educate healthcare providers, patients, and the society at large [51, 52]. It is also imperative to provide all breast cancer patients with psychosocial support in a manner that will be culturally acceptable to them.

### Implications for practice and future research

The findings of this study indicate that all young women did not get adequate support and counseling from their healthcare personnel. The lack of counseling before and after diagnosis worsened the psychological reactions of these young women. As patients’ advocates, nurses should be trained to offer counseling services to women suspected of having breast cancer before and after diagnoses as it is done in the case of Human Immunodeficiency Viral infection in Ghana. Future research could look at developing a Navigation programme for breast cancer care in Ghana and assessing its impact. A quantitative approach could be adopted to look at the impact of psychosocial care in breast cancer patients.

## Conclusion

Breast cancer diagnosis and treatment create physical and emotional problems that interfere with one’s quality of life. Women who are diagnosed with breast cancer require holistic care from healthcare professionals. Every patient suspected of having breast cancer should be counseled before and after diagnosis to psychologically prepare him or her for treatment. It is also necessary to educate young women with breast cancer on the side effects and expectations of the various treatment modalities before their initiation to enable them to cope better.

## Data Availability

The transcripts from which this script was written are available on request from the corresponding author.
